# The effects of cigarette smoking on ventricular repolarization in adolescents

**DOI:** 10.1590/S1679-45082017AO3945

**Published:** 2017

**Authors:** Seyma Kayali, Fadime Demir

**Affiliations:** 1 Keçiören Training and Research Hospital, Ankara, Turkey.

**Keywords:** Adolescent, Smoking/adverse effects, Arrhythmias, cardiac/etiology, Adolescente, Hábito de fumar/efeitos adversos, Arritmias cardíacas/etiologia

## Abstract

**Objective:**

To assess the association between cigarette smoking and ventricular arrhythmias in adolescents. Novel electrocardiographic parameters –Tp-e interval, as well as Tpe/QT and Tpe/QTc ratios – were used to make this assessment.

**Methods:**

The study population consisted of 87 subjects aged between 16-19 years. Fifty-one adolescent smokers with no risk of arrhythmia comprised the Smoker Group, and 36 adolescents who had never smoked cigarettes comprised the Control Group. Smokers were defined as patients smoking more than three cigarettes per day, for at least 1 year. Body mass index, systolic, diastolic and mean blood pressures were measured, and electrocardiograms were performed on all subjects. Heart rate, PR and Tp-e intervals, and Tpe/QT, Tpe/QTc ratio were digitally measured.

**Results:**

Adolescents in Smoker Group had smoked cigarettes for 2.9±1.4 years (range 1 to 6 years). The mean age at starting smoking was 13.8±1.4 years. There were no differences between smokers and Control Group as to baseline clinical variables (p>0.05). The PR, QT and QTc intervals were similar in all groups. Tp-e interval (98.4±12.7ms and 78.3±6.9 ms; p<0.001), Tpe/QT (0.28±0.04 and 22±0.03; p<0.01), Tpe/QTc (0.24±0.03 and 0.19±0.01; p<0.001) ratios were significantly higher in Smoker Group. There were no correlations between years of smoking, number of cigarettes per day, Tpe interval, Tpe/QT or Tpe/QTc ratios.

**Conclusion:**

Cigarette smoking is associated with risk of ventricular arrhytmogenesis with prolonged Tp-e interval and increased Tpe/QT and Tpe/QTc ratios in adolescents.

## RESUMO

**Objetivo:**

Avaliar a associação entre tabagismo e arritmias ventriculares em adolescentes usando novos parâmetros eletrocardiográficos: intervalo Tp-e, e relações Tpe/QT e Tpe/QTc.

**Métodos:**

A população do estudo incluiu 87 indivíduos de 16 a 19 anos de idade. Cinquenta e um adolescentes fumantes, sem risco de arritmia, formaram o Grupo Fumantes, e 36 adolescentes, que nunca tinham fumado cigarros, formaram o Grupo Controle. Os fumantes foram definidos como pacientes que fumavam mais de três cigarros por dia, há pelo menos 1 ano. O índice de massa corporal, e a pressão arterial sistólica, diastólica e média foram medidos, e foram realizados eletrocardiogramas em todos os participantes. Frequência cardíaca, intervalos PR e Tp-e, e as relações Tpe/QT e Tpe/QTc foram medidas por instrumentos digitais.

**Resultados:**

Os adolescentes do Grupo Fumante fumavam há 2,9±1,4 anos (variação 1 a 6 anos). A média de idade ao começar a fumar foi 13,8±1,4 anos. Não houve diferença nas variáveis clínicas iniciais entre os Grupos Fumante e controle (p>0,05). Os intervalos PR, QT e QTc foram semelhantes em todos os grupos. O intervalo Tp-e (98,4±12,7ms e 78,3±6,9ms; p<0,001), e as relações Tpe/QT (0,28±0,04 e 22±0,03; p<0,01) e Tpe/QTc (0,24±0,03 e 0,19±0,01; p<0,001) foram significativamente maiores no Grupo Fumantes. Não houve correlação entre anos de tabagismo, número de cigarros por dia, intervalo Tpe e relações Tpe/QT e Tpe/QTc.

**Conclusão:**

O hábito de fumar está associado ao risco de arritmogênese ventricular, com interval Tp-e prolongado e aumento nas relações Tpe/QT e Tpe/QTc em adolescentes.

## INTRODUCTION

The effects of cigarette smoking on cardiovascular system have been investigated extensively by several studies in adults.^(^ ^1^ ^-^ ^3^ ^)^ These studies demonstrated that smoking is a major cause of atherosclerosis-related cardiovascular events. In addition to this, smoking may also lead to ventricular arrhythmias and sudden cardiac death.^(^ ^4^ ^)^

The influence of smoking on ventricular arrhythmogenesis is explained with ventricular recovery time alterations by limited studies.^(^ ^5^ ^-^ ^8^ ^)^ Nicotine that is released into the circulation during smoking is clearly known to increase the plasma catecholamines, heart rate, and arterial blood pressure and as a result of all those alterations augmented myocardial work and oxygen demand may contribute to the generation of cardiac arrhythmias.^(^ ^9^ ^)^ In an experimental study, Mehta et al.,^(^ ^10^ ^)^ determined the dose-dependent arrhythmogenecity of nicotine in dogs, and reported that higher doses, bioequivalent to smoking two standard cigarettes, can lead to supraventricular arrhythmias, atrioventricular junctional arrhythmias and ventricular arrhythmias. Hayashi et al.,^(^ ^11^ ^)^ compared the influences of nicotine in modulating vulnerability to atrial tachycardia and fibrillation (AT/AF) in young and old rats, and reported that nicotine prolonged the interatrial conduction time and effective refractory period in both groups. They also described that nicotine had biphasic effects on inducible AT/AF in young rats, but suppressed them in the old rats by causing high degrees of interatrial conduction block. In a recent clinical study, Conrad et al.,^(^ ^12^ ^)^ also reported that smoking significantly decreased resting respiratory sinus arrhythmia and increased mean heart rate in adolescents that are the indicators of how well the body maintains homeostasis as a response to environmental demands.

In previous studies, the interval between the peak and the end of T wave (Tp-e) was reported as an indicator of the total dispersion of repolarization, and prolongation of Tp-e was associated with ventricular arrhythmias.^(^ ^13^ ^)^ Moreover, QT interval (QT) and corrected QT interval (QTc) may also show the myocardial repolarization in electrocardiograms (ECG). The Tp-e/QT and Tp-e/QTc ratios are also used as an index of ventricular arrhythmogenesis.^(^ ^14^ ^)^

## OBJECTIVE

To assess the association between cigarette smoking and ventricular arrhythmias in adolescents.

## METHODS

This observational study was approved by the Research Ethics Committee of a public tertiary hospital (number 2012-KAEK-15/1323). The study population consisted of two groups: 51 adolescent smokers were recruited (29 male; mean age 17.2±0.5 years, range 16 to 19 years) for the smoking group (smokers); and 36 healthy subjects who had never smoked before (19 female; mean age 17.05±0.5, range 16 to 18.4 years) were included in the Control Group. Both groups enrolled subjects who had been admitted to the pediatric cardiology department for various reasons (innocent murmur, non-cardiac chest pain, sports participation etc.), between March and June 2016. Smokers were defined as adolescents smoking more than three cigarettes per day, for at least 1 year. Participants were asked not to smoke 30 minutes before the evaluation to avoid the acute nicotine effects. Adolescents who had any risk factors for arrhythmia other than smoking, such as congenital heart disease, rheumatic valvular disease, *diabetes mellitus*, hypertension, abnormal thyroid tests, or who were on medications that could affect ventricular recovery time (such as sertraline, azithromycin, diphenhydramine etc.) were excluded from the study.

The medical history was obtained, and physical and echocardiographic examinations were performed, together with the evaluation of blood pressure and anthropometric data including weight and height, in all participants. Body mass index (BMI) was defined as weight in kilograms divided by the square of height in meters (kg/m^2^). Blood pressure was taken in triplicate after a 10-minute seated rest using a mercury sphygmomanometer and appropriate cuff size. Korotkoff phases 1 and 5 were used to define systolic and diastolic blood pressure, respectively.^(^ ^15^ ^)^ The adolescents diagnosed as presenting any clinical alteration in the physical examination, or cardiac disorder in the transthoracic echocardiography, were also excluded from the study.

An ECG recorder (Nihon Kohden, Tokyo, Japan) set at 25mm/s paper speed and 10mm/mV voltage was used. All ECG were recorded when participants were at rest and on supine position and were transferred to the digital media by scanning with an optical scanner (Hewlett Packard Deskjet 2130). All measurements were digital and taken by one experienced pediatric cardiologist, who was blinded to the smoking status of participants, in order to avoid interobserver variability.

**Figure 1:**
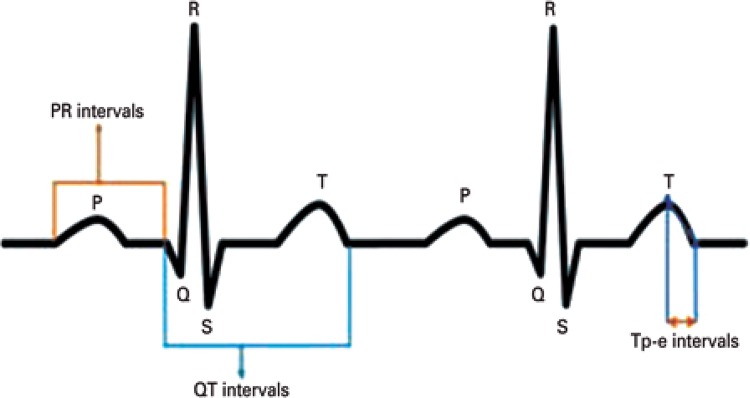
Demonstration of PR, QT and Tp-e intervals in electrocardiogram

The PR interval was measured from the first visible upward of the P wave to the QRS complex starting point (Figure 1). The QT interval was defined from the beginning of the QRS complex to the end of the T wave, and the Tp-e interval, from the peak of T wave to the point it returns to baseline (Figure 1). This point could also be described as intersection of tangent to the slope of T wave and isoelectric line. At least three P waves, T waves, PR intervals, QRS-T complexes were evaluated in each derivation and the mean periods of PR, QT and Tp-e were calculated as milliseconds. The QT intervals were corrected according to Bazett formula, where (QTc=QT/√ RR).^(^ ^16^ ^)^ The Tp-e /QT and Tp-e/QTc ratios were calculated based on these measurements. Rhythm, speed, QRS axis, ventricular hypertrophy and ST-T changes in electrocardiograms were also evaluated.

### Statistical analysis

All statistical analyses were performed using the Statistical Package for the Social Sciences (SPSS) for Windows, version 20.0. Continuous variables were expressed as mean±standard deviation. Kolmogorov-Smirnov test was used to assess the normal distribution suitability of variables. According to distribution, the Student’s *t* test or Mann Whitney U test were used to compare groups. Correlation between variables was assessed by using Spearman’s rank correlation test. A p value of <0.05 was considered significant.

## RESULTS

Clinical characteristics of both groups are summarized in table 1. There was no statistically significant difference between smokers and Control Groups in term of sex, age and BMI. All subjects were in sinus rhythm.

**Table 1:** Clinical characteristics of the study groups*

Characteristics	Nonsmokers n=36	Smokers n=51	p value
Sex, male/female	17/19	29/22	0.39
Age, years	17.1±0.5	17.1±0.6	0.13
Body mass index, kg/m^2^	22.5±2.7	22.1±3.6	0.27
Systolic blood pressure, mmHg	113.7±8.2	116.1±8.9	0.20
Diastolic blood pressure, mmHg	65.3±6.8	69.2±8.6	0.23
Mean blood pressure, mmHg	81.4±6.2	84.8±7.6	0.27
Heart rate, beats/minutes	80±14.7	85.6±10.7	0.053

* Values are presented in mean±standard deviation.

The baseline PR, QT and QTc values were similar in two groups. In the Smoker Group, Tp-e interval was significantly prolonged compared to the Control Group (98.4±12.7msn and 78.3±6.9msn, respectively; p<0.001). A statistically significant difference in Tp-e/QT ratio was also determined between groups (0.28±0.04 and 22±0.03, respectively; p<0.01). In addition to these results, the Tp-e/QTc ratio was also higher in the Smoker Group as compared to controls (0.24±0.03 and 0.19±0.01, respectively; p<0.001) (Table 2).

**Table 2:** Electrocardiogram measurements of the study groups

Electrocardiogram measurements	Nonsmokers n=36	Smokers n=51	p value*
PR, ms	135.1±23	137.7±23.4	0.60
QT, ms	359.9±36.2	346.4±23.6	0.12
QTc, ms	406.1±21.4	411.4±20.17	0.24
Tpe, ms	78.3±6.9	98.4±12.7	<0.01
Tpe/QT, ms	0.22±0.03	0.28±0.04	<0.01
Tpe/QTc, ms	0.19±0.01	0.24±0.03	<0.01

* p<0.05 considered statistically significant.

Adolescents in Smoker Group had been smoking cigarettes for 2.9±1.4 years (range 1 to 6 years). The mean age at starting smoking was 13.8±1.4 years. Twenty-four (47.1%) adolescents in Smoker Group smoked more than ten cigarettes per day. No correlations were determined between years of smoking, number of cigarettes per day and Tp-e interval, or Tpe/QT and Tpe/QTc ratios.

## DISCUSSION

Cigarette smoking increases mortality not only due to induction of coronary artery disease, but also to sudden cardiac death, since smoking is associated with increased sympathetic tone and reduced vagal modulation.^(^ ^4^ ^,^ ^17^ ^,^ ^18^ ^)^ Nicotine is the main component of cigarettes, known as a nonspecific blocker of potassium channels. It has several systemic effects, including tachycardia, increased blood pressure, and catecholamine release; and it is therefore defined as arrhythmogenic.^(^ ^19^ ^,^ ^20^ ^)^

Effects of smoking on ECG were shown in studies that found increased QT and QT dispersion in acute and chronic periods of smoking.^(^ ^7^ ^,^ ^8^ ^)^ In this study, we did not find any differences in the QT and QTc intervals between groups. However, ventricular arrhythmia risk is usually evaluated using QT interval and changes in T wave, and measurements of these changes were also reported as predictor of arrhythmia. The Tp-e interval between the peak and the end of T wave is a reflection of transmural dispersion of repolarization. Prolongation of this interval is a period of potential vulnerability to re-entrant ventricular arrhythmias.^(^ ^13^ ^,^ ^14^ ^)^ In previous studies, an association between increased risk of mortality and prolongation of Tp-e interval was shown in Brugada syndrome, long QT syndrome and hypertrophic cardiomyopathy.^(^ ^21^ ^-^ ^23^ ^)^ A case control study by Panikkath et al., also reported that prolongation of Tp-e interval was associated with sudden cardiac death in general community.^(^ ^24^ ^)^

The effects of cigarette smoking on Tp-e interval were evaluated in a limited number of studies, particularly in adults. İlgenli et al., showed prolongation of Tp-e interval and Tp-e /QT, Tp-e/QTc ratios in long term heavy smokers.^(^ ^6^ ^)^ Taşolar et al., also found QT prolongation and dispersion, in addition to these parameters.^(^ ^5^ ^)^

In this study, the Tp-e interval, as well as the Tp-e/ QT and Tp-e /QTc ratios were found significantly higher in Smoker Group. However, statistically significant difference could not be found by means of QT and QT dispersion. Nonetheless, it seems that this significant difference in Tp-e interval in the Smoker Group could be associated with indirect assessment of repolarization time dispersion. The Tp-e/QT ratio is considered a more sensitive index of arrhythmogenesis, since it is not affected by variations in body weight and heart rate.^(^ ^14^ ^)^ To the best of our knowledge, this study is the first that has determined alterations of Tp-e interval in adolescent smokers.

Akbarzadeh et al., reported that even a single cigarette induces the potential of sudden cardiac death and arrhythmia by QT dispersion prolongation.^(^ ^8^ ^)^ Ilgenli et al., found Tp-e interval, and Tp-e/ QT and Tp-e/QTc significantly higher just after smoking.^(^ ^6^ ^)^ In our study, in accordance with previous studies, cigarette smoking altered ventricular repolarization, regardless of past history. We did not find a statistically significant relation between years of smoking, number of cigarettes per day, and ventricular repolarization parameters. These findings also suggest that cigarette smoking may increase the risk of sudden cardiac death even in adolescents, due to alterations in ventricular repolarization, apart from the cumulative and long-term effects.

Cigarette smoking is known to increase the sympathetic outflow and heart rate.^(^ ^25^ ^)^ In the present study, we also determined that the mean heart rate of smokers was higher than the Control Group, but the difference was at significance margin (p=0.053). In this study, we asked participants not to smoke for, at least, half an hour before evaluation in order to avoid the acute effects of nicotine, which may alter heart rate and increase QT and QTc measurements. We did not observe any difference regarding QT and QTc measurements between groups, and the reason may be the increased heart rates that were positive at statistical margin. It is well known that smoking is a worldwide public health problem. Rosewich et al., reported the addiction to nicotine develops mainly before the age of 20 years (80% of all adults smokers started as teenagers) in Germany, and the proportion of adolescent smokers has regularly increased in recent years.^(^ ^26^ ^)^ In the present study, the mean age at starting smoking was found to be as young as 13.8±1.4 years and adolescents in Smoker Group had been smoking for 2.9±1.4 years (range of 1 to 6 years).

### Limitations

This study is a preliminary study and has some limitations. First, it included only a small number of participants, and future larger studies may provide more accurate results. Second, we did not ask and record the interval between the last cigarette smoked and the ECG recording in the Smoker Group. Smoking is known to have some acute effects on cardiac functions and participants were asked not to smoke for half an hour before the evaluation in order to avoid those acute effects. Further studies may investigate the effects of smoking on different time periods. And, third, only short-term records could be obtained. Further prospective follow-up studies should be performed to understand the prognostic importance of Tp-e interval prolongation and increased Tp-e/QT ratio in ventricular arrhythmogenesis and sudden cardiac death.

## CONCLUSION

This was the first study in adolescents that has reported prolonged Tp-e interval and increased Tp-e/QT and Tp-e/QTc ratios in smokers. The present study provides evidence that smoking is associated with alterations in ventricular repolarization time that may contribute to ventricular arrhythmogenesis.
